# Supplementary material for: leafkin—An R package for automated kinematic data analysis of monocot leaves
Source: Quant Plant Biol. 2020 Nov 30;1:e2. doi: 10.1017/qpb.2020.3 (PMC10095969; doi:10.1017/qpb.2020.3)
Supplement: Supplementary file 1 [file S263288282000003Xsup001.pdf]

leafkin user manual

Supplementary File S1

An R package for automated kinematic data analysis of monocot leaves.

Preface

Growth is one of the most studied processes in plants. At the cellular level, plant growth is driven by cell division and cell expansion. Kinematic analysis, a method to quantify the contribution of these two cellular processes to organ level growth, has been developed and perfected over the past decades. To streamline the data analysis of the raw data, collected during the lab work involved, we have created ***leafkin***, an R-package to perform all the calculations in the kinematic analysis of monocot leaves using four functions. These functions support leaf elongation rate calculations, creating plots of fitted cell lengths, extraction of fitted cell lengths and execution of all kinematic equations.

The goal of this manual is to provide a step-by-step manual, with attention for technical aspects such as file paths and troubleshooting errors. The manual works closely with a tutorial script and sample data, which can be downloaded following the instructions in this manual.

Table of Contents

Preface 2

Table of Contents 3

Kinematic analysis summarised 4

A brief introduction to the practical work involved in a kinematic analysis of monocot leaves 5

Flowchart 6

Requirements 7

- Required data files 7
- R and RStudio installation 9
- Download sample data and tutorial script 10
- Install *leafkin* 13

Kinematic data analysis using *leafkin* 14

- Step 1: Load your data / file paths 14
- Step 2: Calculating the average leaf elongation rates 16
- Step 3: Inspect the cell length fits 17
- Step 4: Obtaining the fitted cell lengths 20
- Step 5: Performing the kinematic analysis calculations 21
- Additional code 23

Errors and troubleshooting 24

- Installation of *leafkin*. 24
- Extra columns could be added through the use of Excel when creating the .txt file. 24
- Error when creating the pdf containing cell length fit plots: cannot open file 24
- Error when fitting cell lengths related to gridsize: Binning grid too coarse for current (small) bandwidth. 24

Additional information 25

- Tidy and wide data 25
- Tibbles 26

Formulas used by *leafkin* 27

References 30

Kinematic analysis summarised

Practical work

- **Growth measurements**: Grow plants and track the growth of the leaf of interest by making daily leaf length measurements.
- **Microscopy study**: For a set of plants, usually around 5-7, a microscopy study is performed on leaf material collected during steady state growth (i.e. while leaf elongation rate is constant). Cell length data collected during the microscopy study will be combined with the leaf length measurements in the data analysis.
- **Create data files**: After having collected the measurements, create the data files containing your raw data. **Please pay attention to the structure and units of the data (see data files section), since these are fixed.** Wrong units will result in erroneous conclusions.

Leafkin installation

- Install *leafkin* from the impres-lab GitHub page (https://github.com/impres-lab). For installation instructions, please consult the *leafkin* installation section of the manual.
- On the impres-lab GitHub page, a tutorial script (incl. code for leafkin installation) can be downloaded, together with sample data files. **We *highly* recommend first time users to use the tutorial script and data for leafkin installation and to familiarize yourself with the functions and data structure.**

Data analysis using leafkin

Kinematic data analysis using *leafkin* takes place in **four distinct steps**, each represented by a specific function of leafkin:

1. ***calculate_LER()***: this function uses the *leaf length measurements* to calculate the average leaf elongation rates for each plant over a (by the user) specified number of calculated leaf elongation rates. These average leaf elongation rates will be used in the kinematic_analysis() function.
2. ***get_pdf_with_cell_length_fit_plots()***: this function uses the *cell length measurements* and creates a pdf containing plots of the cell length fits. This pdf allows you to evaluate the fit using different settings. The function contains a bandwidth multiplier parameter which can make the fit stricter or more loose.
3. ***get_all_fitted_cell_lengths()***: this function also uses the *cell length measurements*, but this time, it extracts all fitted cell lengths for a given interval. The fitted cell lengths are used in the *kinematic_analysis()* function.
4. ***kinematic_analysis()***: this function performs all calculations involved in a kinematic analysis, using data from your previous calculations (i.e. *average leaf elongation rates* and *fitted cell lengths*), together with *meristem measurements*.

Data presentation and statistics

Running the *kinematic_analysis()* function generates all kinematic parameters for each plant included in the analysis. These data can then be statistically analysed and presented in graphs and/or tables.

Done

A brief introduction to the practical work involved in a kinematic analysis of monocot leaves

For those unfamiliar with the practical work involved in a kinematic analysis, we provide a brief introduction on the practical work involved. To fully understand each step and execute it with attention for the details, a more extensive protocol can be consulted (Sprangers *et al.*, 2016). Besides detailed written instructions, Sprangers *et al.* (2016) provides a video tutorial illustrating each step of the kinematic analysis.

Practical work involved in a kinematic analysis of monocot leaves:

1. Grow your monocotyledonous species of interest and select a leaf to study. In maize seedlings, the IMPRES lab (Antwerp University, Belgium) works on the fifth leaf to limit the duration of the experiment (ca 3 weeks), while the growth is no longer supported by seed reserves and treatments have sufficient time to establish and impact growth.
2. As soon as the leaf of interest emerges, measure it daily with a ruler (in mm). In our experience with maize, the fifth leaf is growing at a constant rate for at least three days from the time it emerges from the whorl of older leaves (steady state growth).
3. Three days after emergence, during the steady state growth, dissect the plant and isolate the entire leaf of interest form the other leaves. Pay special attention for the younger leaves already growing inside the leaf of interest. These need to be removed as well.
4. For the leaf of interest, retain the basal 11 cm, i.e. the part which was attached to the stem. One side of the blade (lateral of the mid vein) is used for cell length measurements, while the other side is used for meristem size measurements.
    a. For the meristem measurement, a 3 cm segment from the basal part, i.e. starting from where the leaf wat attached to the stem, is retained and stored in a 3:1 (v:v) absolute ethanol:acetic acid solution for at least 24h at 4 °C. Through DAPI staining and fluorescence microscopy, mitotic figures are visualised in the epidermis. The most distal mitotic figure (i.e. the one furthest away from the base of the leaf) is used as a reference for the meristem border. The distance from the leaf base to this mitotic figure is the meristem size (stored in µm for each plant).
    b. For the cell length measurements, the other half of the leaf is used. It is first stored in denatured absolute ethanol at 4 °C to remove pigments. The absolute ethanol can be replaced several times in order to obtain a cleared leaf. When the leaf is as clear as possible, the absolute ethanol is replaced by pure lactic acid (wear gloves), which will make the leaf tissue flexible again (ethanol treatment makes it brittle). The leaf can be stored in the lactic acid at 4 °C for several months if needed. The first 10 cm of the 11 cm leaf is now dissected into 10 one-centimetre segments, in which cell length measurements are made (in µm). Dissecting the tissue in 10 one-centimetre segments will facilitate navigating and positioning your lens across the leaf, which it is less easy when using longer segments. Using a microscope equipped with differential interference contrast optics allows for visualisation of the cell walls. The cell walls can then be used to measure the cell size across the first (basal) 10 cm of the leaf.

Leaf length, meristem size and cell length measurements provide the input for the data analysis with *leafkin*.

Flowchart

Below, the relation between de data and the functions is shown. On the top, the raw data is presented in dark blue boxes. They are fed to functions, presented in the grey boxes. These functions produce the intermediate data in the light blue boxes, which can then be fed in the *kinematic_analysis()* function at the bottom to perform the final calculations and obtain the results for all the kinematic parameters for each plant. The dotted line and the green box represents a conclusion which can be made on the cell length fit plots in order to continue safely with the *get_all_fitted_cell_lengths()* function.

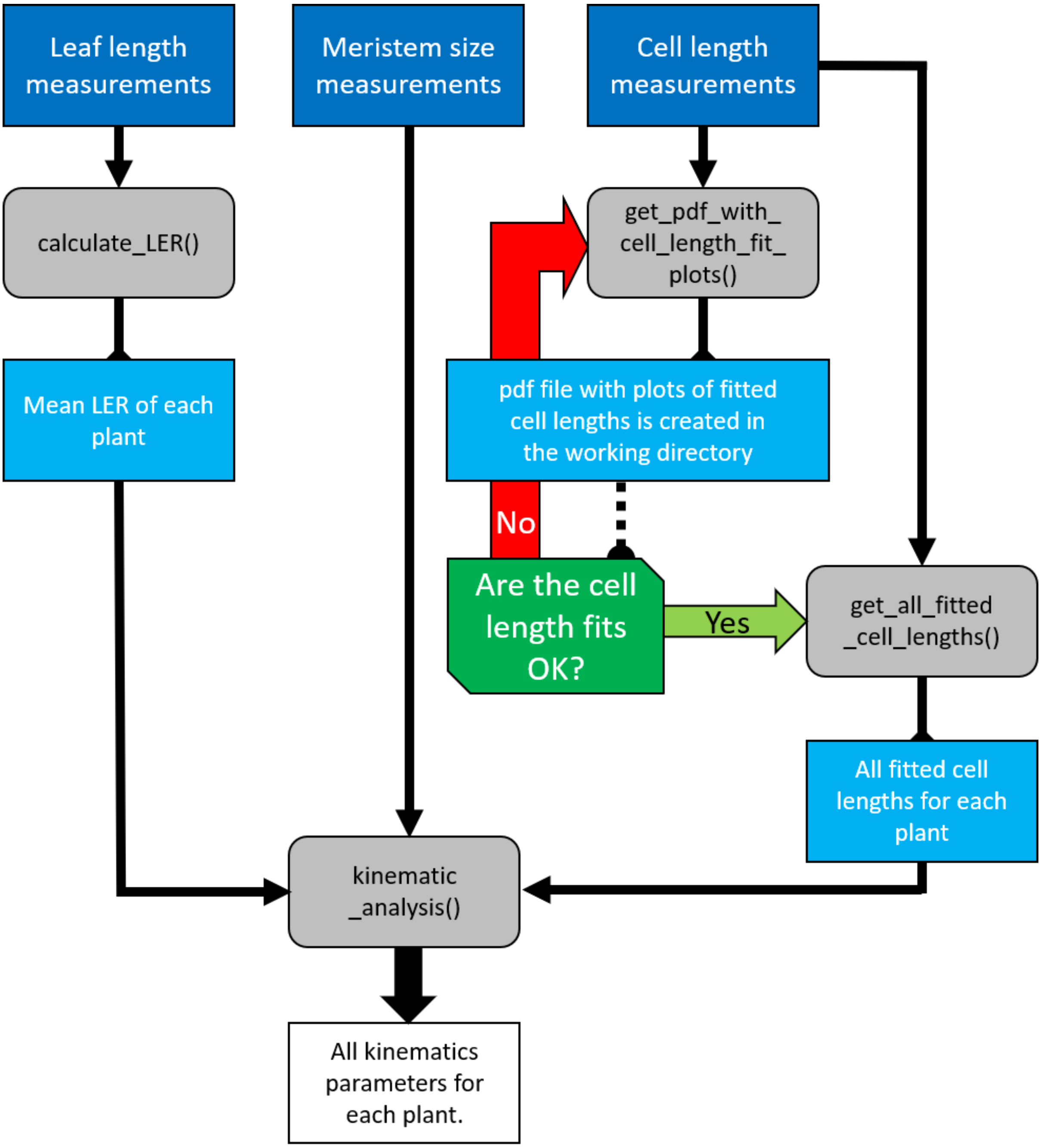

Requirements

The next sections contain information on the requirements in order to use the *leafkin* library:

- Required data files
- Required software
- *Leafkin* installation

Prior to *leafkin* installation, downloading the sample data and tutorial script is described. The tutorial script contains code to install *leafkin*.

Required data files

As introduced in the kinematic analysis overview, three datasets with raw data should be available after performing the practical work:

- Leaf length measurements
- Cell length measurements
- Meristem size measurements

We advise to prepare the raw data in a spread sheet program (e.g. Microsoft Excel), using the provided files as a template. The raw data should be saved as tab-delimited text files. We advise to use this format because importing Excel files directly into R transforms date-times into numbers and renders them unusable by the *leafkin* functions.

The data files should be structured in the following way:

- The leaf length data file (sample data: growth_measurements_millimetre.txt) requires a column with unique plant ID's, followed by multiple columns containing leaf length measurements, expressed in millimetres (Table 1A). The first row contains the headers, which should be *plant_id* for the first column, while the following column headers are in the date-time format *yyyy/mm/dd hh:mm* (or *yyyy/mm/dd hh:mm:ss*), indicating when measurements were made.
- The cell length measurements (sample data: cell_length_measurements_micrometre.txt) are organised in three columns (Table 1B). The first column (header = *plant_id*), holds the plant ID for each measurement. The second column (header = *position*) contains the position of the cell length measurements relative to the leaf base (in centimetres) and is followed by the cell lengths themselves (in micrometres) in the third column (header = *cell_length*). Cell length measurements of all plants are combined in these three columns.
- The third file (sample data: meristem_size_micrometre.txt) contains the meristem size measurements (Table 1C). The first column (header = *plant_id*), contains the unique plant ID's, whereas the second column (header = *mer_length_um*), contains meristem sizes (in micrometres).

Important notes:

- Units and column names should be strictly respected. Wrong units = Wrong conclusions
- Plant ID's should be identical across all three files since these are used to combine the data originating from the different measurements.
- When entering data, do not use NA or any other remark to replace missing data. Keep cells with missing data completely empty.

Table 1. Example data and column description for the datasets required for kinematic analysis using *leafkin.* A: Example of leaf length measurements data and column descriptions. B: Example of cell length measurements data and column descriptions. C: Example of meristem length measurements data and column descriptions. The types char, int and double refer to respectively characters (i.e. everything which includes letters, or numbers specified to be handled as letters), integers (i.e. numbers without decimals) and double (i.e. numbers which can contain decimals).

A. Leaf length measurements data and column descriptions

Example data

plant_id	2016/12/13 10:00	2016/12/14 10:00	2016/12/15 10:00	2016/12/16 10:16	2016/12/17 10:00
C.1	142	216	293		
C.2		142	212	296	
C.3		196	277	360	436
C.4		194	268	352	
…					

Column description

COL	HEADER	TYPE	BRIEF DESCRIPTION
**1**	plant_ID	char or int	Contains the plant ID for which leaf lengths were measured.
**2-LAST**	data time format yyyy/mm/dd hh:mm(:ss)	int or double	Contains leaf length measurements in millimetre on a certain day-time. Time can be in hh:mm or hh:mm:ss.

B. Cell length measurements data and column descriptions.

Example data

plant_id	position	cell_length
C.1	0.01	27.18
C.1	0.01	23.71
C.1	0.01	23.68
C.1	0.01	22.23
…	…	…

Column description

COL	HEADER	TYPE	BRIEF DESCRIPTION
**1**	plant_ID	char or int	Contains the plant ID for which cell lengths were measured.
**2**	position	int or double	Contains the position at which cell lengths were measured in centimetre.
**3**	cell_length	int or double	Contains cell length measurement in micrometre.

C. Meristem length measurements data and column descriptions.

Example data

plant_id	mer_length_um
C.1	12423
C.2	14792
C.4	12350
C.7	14568
…	…

Column description

COL	HEADER	TYPE	BRIEF DESCRIPTION
**1**	plant_ID	char or int	Contains the plant ID for which meristem lengths were measured.
**2**	mer_length_um	int or double	Contains the length of the meristem in micrometre.

R and RStudio installation

Leafkin requires the following to be installed:

- R, version 4.0.0 or higher.
    - The most recent version of R can be installed from the CRAN website, https://cran.r-project.org/.
    - Users already using an older version of R need to update to version 4.0.0 or higher.

We also strongly advise to install:

- RStudio
    - RStudio provides a graphical user interface which makes the use of R more convenient. The tutorial also assumes RStudio to be installed. Do note that its installation does not install R as well. For it to work, R must also be installed. With R and RStudio both installed, make sure to work within RStudio. You do not need to open R, since RStudio will take care of R for you.
    - A free open source edition of RStudio is available on https://rstudio.com/ (RStudio Team, 2015).
- Rtools40 (for Windows users)
    - Local compilation of *leafkin* on Windows systems will result in a warning when Rtools40 is not installed.
    - Rtools40 can be downloaded here: https://cran.r-project.org/bin/windows/Rtools/

Download sample data and tutorial script

FROM HERE AND ONWARD, THE USER MANUAL WILL FOLLOW A TURORIAL SCRIPT AND USE SAMPLE DATA AVAILABLE FROM THE IMPRES-lab GitHub page: https://github.com/impres-lab

The sample data files and tutorial script can be downloaded all together from the leafkin-sample-data-and-tutorial-script repository as instructed here below. The data provided in as sample data originates from a recent publication in which a kinematic analysis was performed (Bertels *et al.*, in press).

Note that the content present on the print screens below might not match the current version.

On the impres-lab GitHub, open de leafkin-sample-data-and-tutorial-script repository.

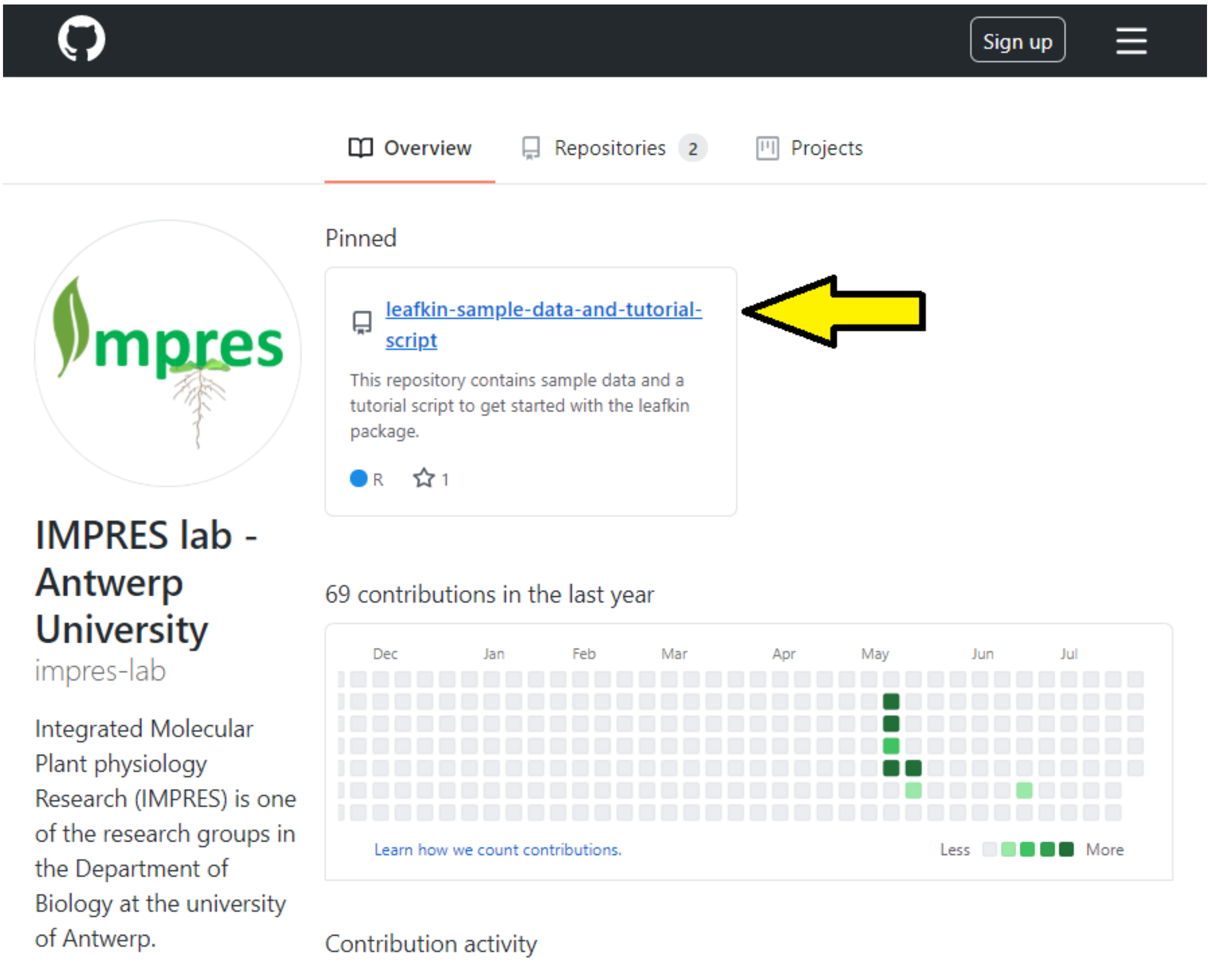

Within the leafkin-sample-data-and-tutorial-script repository, download its entire contents as a zipped folder. You can do this by clicking on the drop-down menu “Code” and select “Download ZIP”:

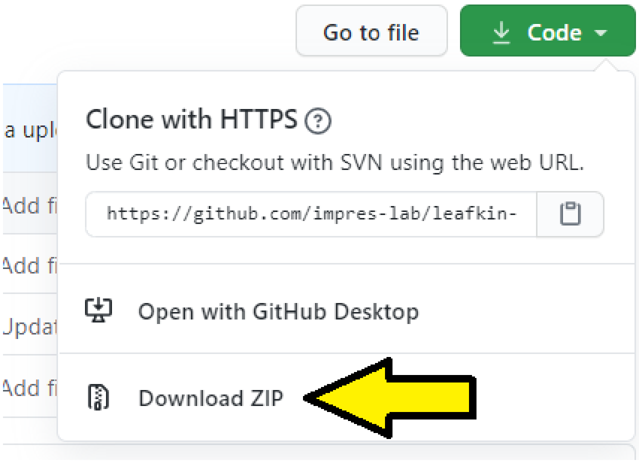

When downloaded, extract the zipped folder. **It is important that the folder is extracted**. Double clicking a zipped folder might allow you to view its contents, but will not allow proper functioning of the script which it contains.

Within the downloaded folder, the following files are present:

- An RStudio project file (.Rproj extension)
  - This file should be opened in RStudio (which should launch by default when trying to open this file).
- An R-script (.R extension)
  - From within the RStudio (with the project file opened therein), this R-script can be opened.
- A README file (.md extension)
  - This file contains information. Its primary function is to provide information on the GitHub repository page.
- A data files folder
  - This folder contains the sample data. Both the Excel files and the txt files are provided. Note that the txt files (tab delimited) are used by the R script. The excel files are just there to serve as an example.
    - cell_length_measurements_micrometre.txt
    - cell_length_measurements_micrometre.xlsx
    - growth_measurements_millimetre.txt
    - growth_measurements_millimetre.xlsx
    - meristem_size_micrometre.txt
    - meristem_size_micrometre.xlsx

When the files are extracted, open the R-project-file (.Rproj extension). This will automatically open RStudio for you. Within the Files tab (on the bottom right), you can now see the contents of the working directory. If you click on the R SCRIPT file, the R-script will be opened in RStudio:

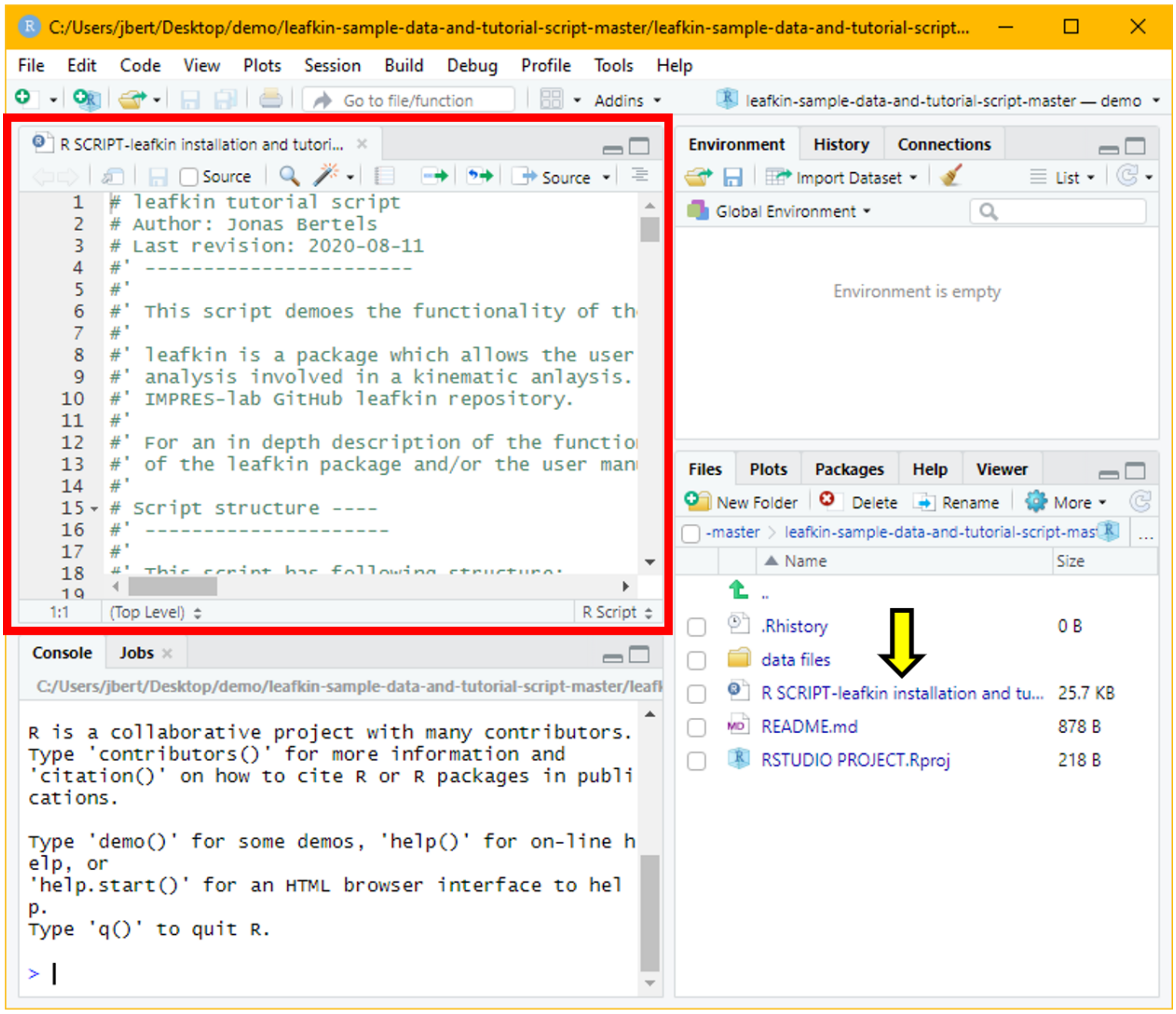

With the tutorial R script opened in the RStudio Project, the user is ready to install *leafkin* and to perform the kinematic data analysis. The tutorial script contains a quick start, containing all the steps of a kinematic data analysis with minimal explanation. This quick start is followed by a more elaborate code section, where each step of the kinematic analysis is addressed in more detail. At the end of the script, we highlight some extra functionalities.

Working with an R-project has a major advantage. The folder where the R-project file is positioned in, is automatically set up as the working directory. This means that R will look in this folder for files to use or that R will create files here when you order it to.

Explaining how to work within RStudio is beyond the scope of this manual, but, for those new to RStudio, the How To R YouTube channel has a nice video introduction on how to work within RStudio:
https://www.youtube.com/watch?v=lVKMsaWju8w

When you want to learn more about R, RStudio Education is a nice place to start:
https://education.rstudio.com/learn/beginner/

Install *leafkin*

Prior to installing *leafkin*, we install the tidyverse and devtools packages by running the following line of code:

```
install.packages(c("tidyverse", "devtools"))
```

The devtools package allows installing *leafkin* from the impres-lab GitHub page. The tidyverse is a collection of packages which are used by the tutorial script as well.

Next, we can install *leafkin*. During the intallation of *leafkin* using the following function, you might be prompted to install and/or update the packages used by the *leafkin* package. On you first try, you should choose to install/update all packages suggested by the function (first option, **1: All**, when prompted: "Enter one or more numbers, or an empty line to skip updates:").

- If, during installation, you are prompted with the question to compile packages or not (i.e. "Do you want to install from sources the package which needs compilation? (Yes/no/cancel)"), the most stable option here is to choose **no**, since local compilation of the latest uncompiled packages sometimes results in an error. When you select **no**, the latest precompiled version of the packages is installed, which is just fine for *leafkin*.

- When you have tried this and there is still an error occurring during installation, you should skip updates of the remaining packages causing an error during installation (just hit your **ENTER** key when prompted: "Enter one or more numbers, or an empty line to skip updates:". This should install *leafkin*.

Run this line to install *leafkin*:

```
devtools::install_github("impres-lab/leafkin")
```

With *leafkin* and the tidyverse packages installed, we can now load the functions into the current RStudio session to make all of their functions available for use. Do this by running the following two lines:

```
library("leafkin")

library("tidyverse")
```

You are now ready to perform kinematic data analysis.

Kinematic data analysis using *leafkin*

For the manual, we will use the code in the quick start section of the sample script, but feel free to run the code with the more elaborate explanation in the second part of the script as well.

Step 1: Load your data / file paths

First, the data is loaded into our R session. The data provided in as sample data originates from a recent publication in which a kinematic analysis was performed (Bertels *et al.*, in press). For this, we need to do two things for each file:

1. Create file path which to tell R where to find the files.
2. Give this file path to the *read_tsv()* function which will read the contents of the file and make it available in R.

To create the file path, we use the *file.path()* function to help us and store the created file path in a variable. For example, in our extracted folder, we find the data files folder. Within the data files folder are the data files we need to read into the R sessions. As mentioned previously, RStudio set the folder from which the R project file is opened as the working directory. Within the working directory, we want R to look for the data files in the data files folder. Therefore, we create the file path where we first have “data files”, followed by the name of the file, for example “growth_measurements_millimetre.txt”. The path is stored within a variable, different for each file. This variable is then used in the *read_tsv()* function to read the data from the files into the R session.

Running these lines will import the contents of the files:

```
# 0.3 Load required data into R
#' Leaf length data
leaf_length_measurements_path <- file.path("data files",
                                           "growth_measurements_millimetre.txt")
leaf_length_measurements <- read_tsv(leaf_length_measurements_path)

#' Cell length data
cell_length_measurements_path <- file.path("data files",
                                           "cell_length_measurements_micrometre.txt")
cell_length_measurements <- read_tsv(cell_length_measurements_path)

#' Meristem size data
mersitem_length_measurements_path <- file.path("data files",
                                               "meristem_size_micrometre.txt")
meristem_size <- read_tsv(mersitem_length_measurements_path)
```

Of course it is possible to create file paths to any file on your computer. For this, we also advice to use the *file.path()* function, since file paths are operating system specific and the *file.path()* function automatically makes a path in the right format.

Looking into the details of a file on both Windows and MacOS systems will reveal you the file path. On MacOS, you can also easily copy the file path by right-clicking the file in the finder while holding down the option key. In the dropdown menu, the option to copy as pathname is now revealed. This file path can be used directly in R. Using Windows file paths directly in R is not possible since they have another format, we therefore advice to recreate the path yourself using the *file.path()* function.

After importing the data, you can see that the data is now imported in the Environment tab on the top right:

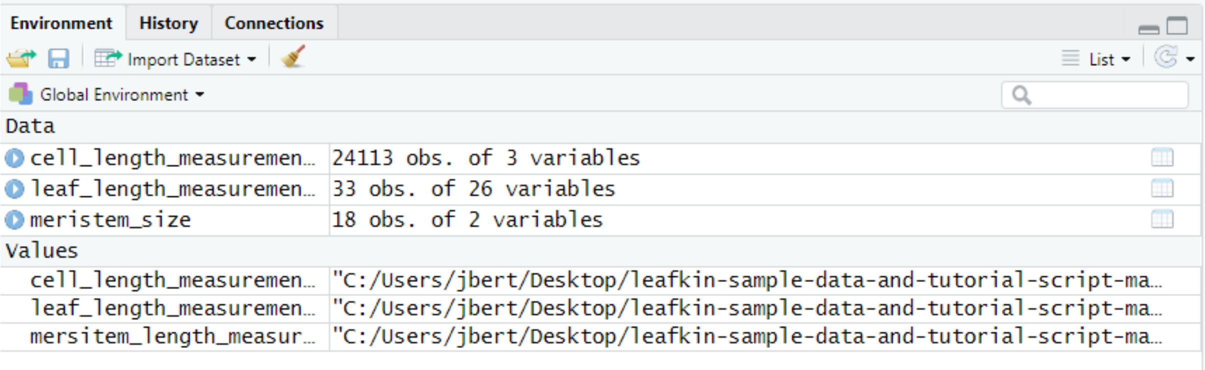

Step 2: Calculating the average leaf elongation rates

Next, we calculate the average leaf elongation rates during the interval of steady state growth and prior to the harvest of the leaves of each plant using the *calculate_LER()* function. We store the result in the *result_LER_means* variable.

```
# 0.4 Perform kinematic analysis
# 0.4.1 Average leaf elongation rates of each plant
result_LER_means <- calculate_LER(leaf_length_data = leaf_length_measurements,
                                  n_LER_for_mean = 2,
                                  output = "means")
view(result_LER_means)
```

For the first parameter of the function, we indicated that the *leaf_length_data* are stored in the *leaf_length_measurements* table. For the second parameter, i.e. *n_LER_for_mean*, we want the first two calculated leaf elongation rates (prior to the harvest of these leaves, for the cellular analysis below) to be used for the calculation of the mean. The last parameter determines the output, which in the kinematic analysis should be means. Notice the parenthesis around means: “means”. This is because means should be interpreted as text. If you do not use the parenthesis, R will not know what you are referring to (since we have not made a variable called means).

The view(*result_LER_means*) will open the results for you within RStudio, allowing you to inspect them visually. Notice that an extra line has appeared in the Environment tab of RStudio, now also including *result_LER_means*.

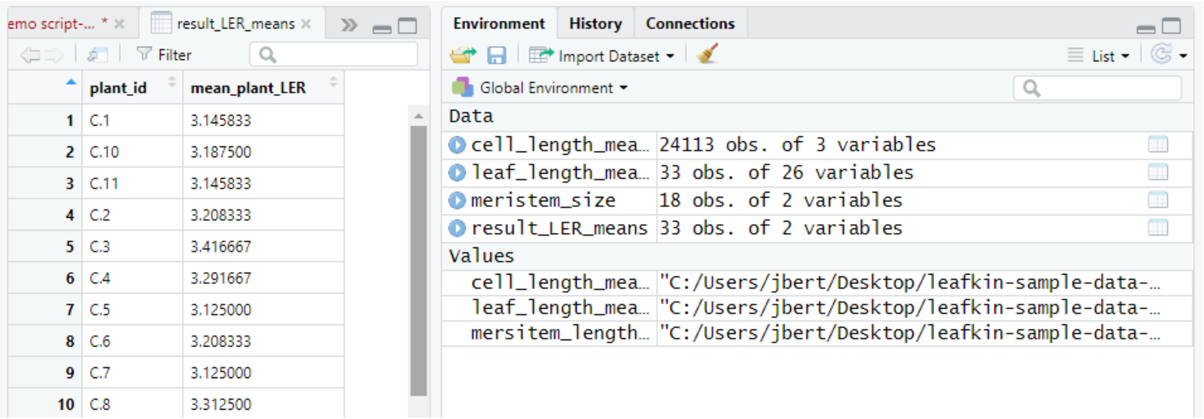

Step 3: Inspect the cell length fits

The *get_pdf_with_cell_length_fit_plots()* function will create a pdf file in your working directory with plots of fitted cell lengths. Also plots of the derivative of this curve will be printed into the pdf, visualising the slope of the fit.

Within this function, we indicate that the *cell_lenght_data* can be found in the *cell_length_measurements* parameter. Next, we set the *interval_in_cm* parameter to 0.1 for cell lengths to be estimated every 0.1 cm. In addition, we can control the fit of the function by setting the *bw_multiplier*. By default, the *bw_multiplier* is set to 1, which means that the calculated bandwidth is multiplied by 1 and thus not manipulated. We also want the function to return a tibble (see additional information for tibbles at the end of the manual) containing the calculated bandwidths. For this, we set *output_bw_tibble* to TRUE. These bandwidths can be used to provide an alternative bandwidth for plants which had a failed bandwidth calculation in the next step (step 4: Obtaining the fitted cell lengths).

```
# 0.4.2 Evaluate cell length fits and store bandwidths
#' A pdf file with the cell length fits will be created in the working directory.
bw_tibble <- get_pdf_with_cell_length_fit_plots(cell_length_data = cell_length_measurements,
                                                interval_in_cm = 0.1,
                                                bw_multiplier = 1,
                                                output_bw_tibble = TRUE)
```

After running this line, a pdf file with the fits has appeared in the working directory:

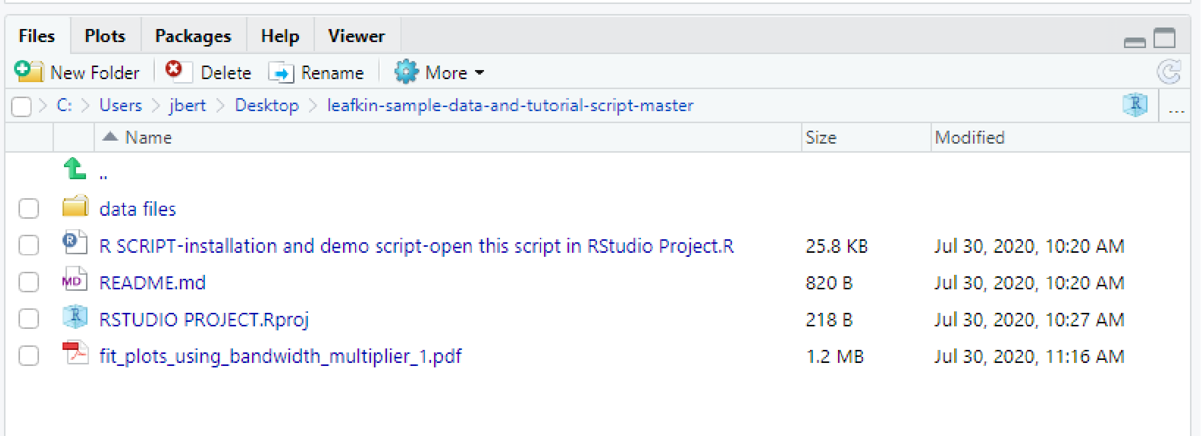

The created pdf-file with the plotted cell lengths and fit curves can be used to evaluate the cell length fits for each plant and to check the impact of set bandwidth multipliers on these fits. A good fit does not overly follow minor local variations in cell length, but closely fits the global profile. When the *bw_multiplier* value was set too low, e.g. 0.3, too much local variation could be introduced in the fit, especially in the mature region, where cell length can be considered approximately constant (Figure 1A). Inversely, when the *bw_multiplier* value was set too high, e.g. 3, oversmoothing could occur, particularly affecting fitted cell sizes in the meristem.

In the created pdf file, also the calculated bandwidths for each individual plant are plotted in the last graph. If, for some plants, the function was unable to calculate the optimal bandwidth (for example when an insufficient number of cell length measurements was provided), there will be missing data in the bandwidth plot, but also will the concerned cell length fit plots have no fit and a warning message will be printed in the console of RStudio. In this case, when extracting all the fitted cell lengths in the next step, an alternative bandwidth should be provided in the *get_all_fitted_cell_lengths()* function (see next section).

The effect of the bandwidth parameter on cell length fits:

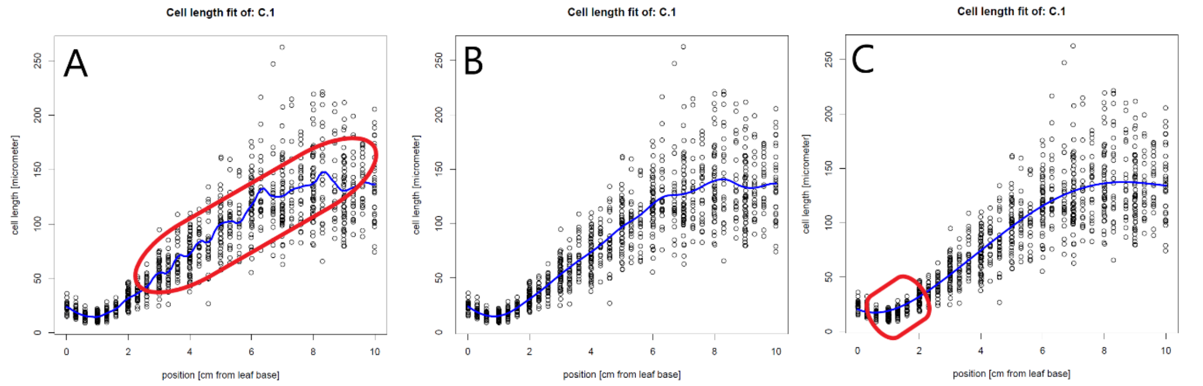

A) A very strict fit of the cell lengths by setting the *bw_multiplyer* to 0.3. A strict fit can result in too much variation (encircled in red). **B)** Fitted cell length data, using the calculated bandwidth (bandwidth multiplier = 1). **C)** A more loose fit of the cell lengths by setting the *bw_multiplyer* to 3. A loose fit can result in oversmoothing, poorly fitting cell sizes, especially at the end of the meristem (encircled in red) and/or the end of the growth zone.

If the fit is not good, vary the *bw_multiplier* parameter and asses each fit until you have found the most satisfying fit. Your ideal *bw_multiplier* value can then be used in the next step, where we extract the fitted cell lengths.

Extra:

In addition to the cell length fit plots, plots of the local first derivative (i.e. slope) are also printed (where in the y-axis label, dl/dx, l = cell length and x = position). These can be used to assess the smoothness of the fit.

The effect of the bandwidth parameter on the slope of the cell length fits:

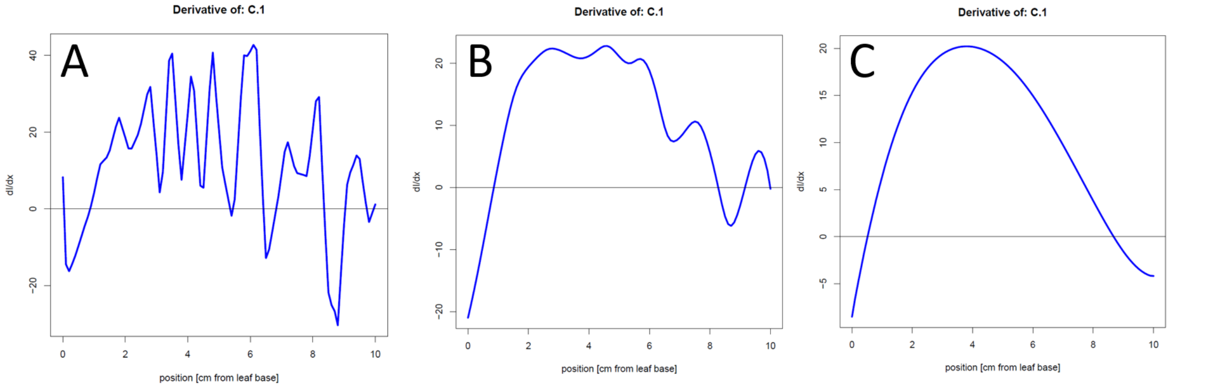

A) A very strict fit of the cell lengths by setting the *bw_multiplyer* to 0.3 results in a lot of variation in the slope. **B)** The unmanipulated calculated bandwidth (bandwidth multiplier = 1) resulted here in an acceptable amount of variation in the slope. **C)** A more loose fit of the cell lengths by setting the *bw_multiplyer* to 3 resulted in a slope which does not follow any variation in the data.

The last graph of the document, contains all calculated bandwidths:

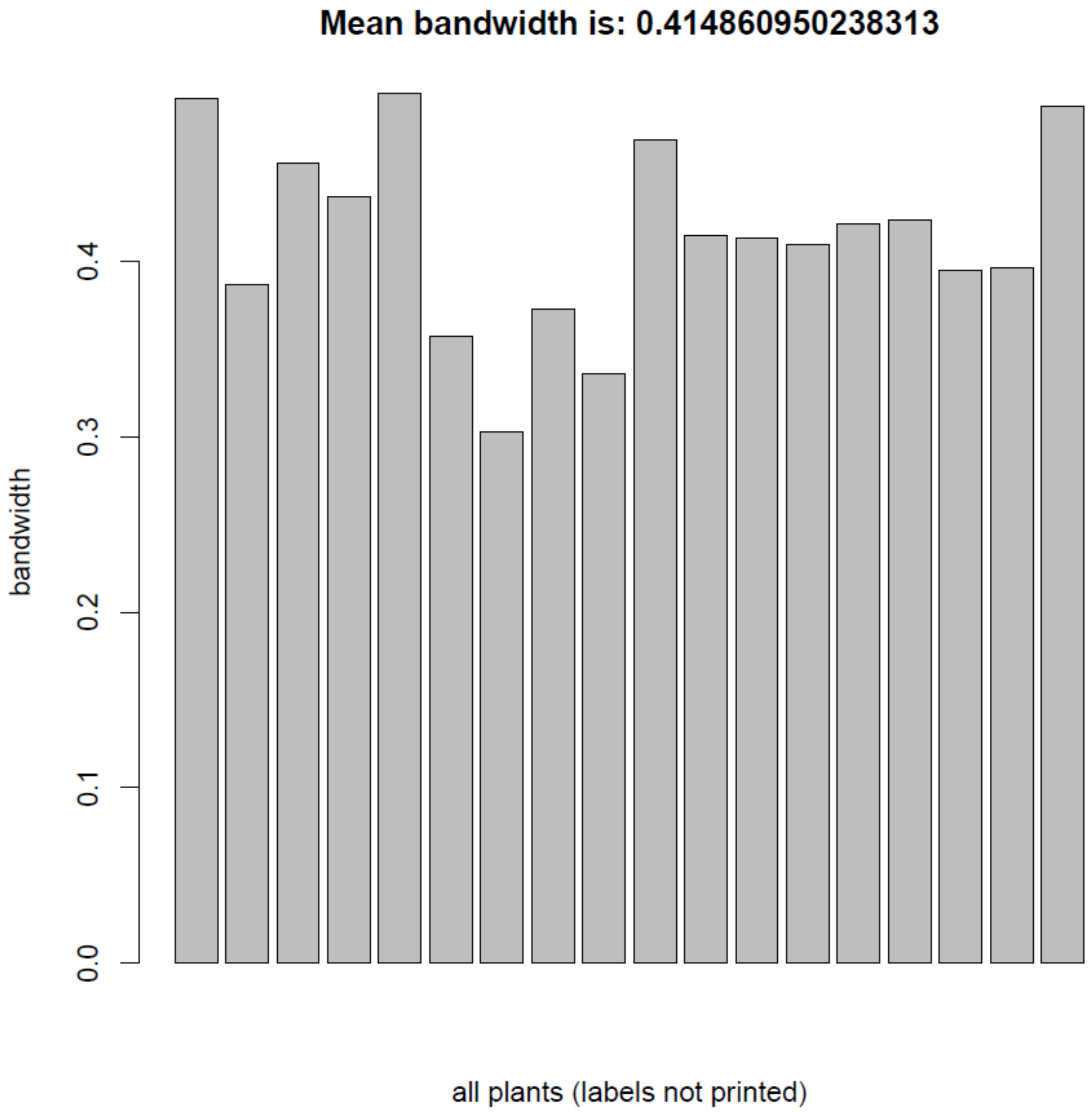

If, for some plants, the function was unable to calculate the optimal bandwidth (for example when an insufficient number of cell length measurements was provided), there will be missing data in the bandwidth plot, but also will the concerned cell length fit plots have no fit and a warning message will be printed in the console of RStudio.

Step 4: Obtaining the fitted cell lengths

During the microscopy study, it is impossible to obtain cell lengths at every possible location. However, a fit of the measured cell lengths will allow to obtain estimated cell lengths at every desired location.

With the *get_all_fitted_cell_lengths()* function, we obtain the fitted cell lengths for a user-set interval and store them in the *fitted_cell_lengths* variable.

```
# 0.4.3 Obtain fitted cell lengths.
fitted_cell_lengths <- get_all_fitted_cell_lengths(cell_length_data = cell_length_measurements,
                                                   interval_in_cm = 0.1,
                                                   bw_multiplier = 1,
                                                   alternative_bw = mean(bw_tibble$collected_bandwidths,
                                                                         na.nm = TRUE),
                                                   tidy_cell_lengths = TRUE)
```

As you can see, this function again requires you to supply the *cell_lenght_measurements* data to the *cell_length_data* parameter. Next, we set the interval at which cell lengths should be obtained, in this case every 0.1 cm. The bandwidth does not have to be manipulated, so we set the multiplier to 1 (this is the default value). All of our plants had a calculated bandwidth, but for the sake of completeness, we added here a line which calculates the mean of the *collected_bandwidths*, present in the *bw_tibble* (where na.rm = TRUE removes missing bandwidth values). Lastly, the most important line is the way we want the fitted cell lengths to be returned. It is important that they are returned in a tidy format (see addition information at the end of the document for more information on the tidy format), so we set *tidy_cell_lengths* to TRUE.

Inspecting the *fitted_cell_lengths* will show you the estimated cell length (µm) at every position (cm) of the given interval. Reducing the interval size is possible of course.

	plant_id	position	cell_length
1	C.1	0.0	23.93619
2	C.1	0.1	21.96697
3	C.1	0.2	20.19933
4	C.1	0.3	18.64897
5	C.1	0.4	17.33900
6	C.1	0.5	16.27782
7	C.1	0.6	15.48228
8	C.1	0.7	14.97352
9	C.1	0.8	14.73420

With the fitted cell lengths collected, we can now move on to the kinematic analysis calculations.

Step 5: Performing the kinematic analysis calculations

With the *kinematic_analysis()* function, the all the kinematic analysis calculations can be executed at once for every plant in the microscopy analysis. The results of the average leaf elongation rate calculations are supplied to the *LER_means* parameter, the fitted cell lengths are supplied to the *tidy_cell_lengths* parameter and the meristem sizes, which were imported as a simple table in the beginning, are assigned to the *meristem_size_micrometre* parameter. The result is stored in the *final_kinematic_analysis* variable.

```
# 0.4.4 Perform kinematic analysis
#' Remaining kinematics calculations, all at once.
final_kinematic_analysis <- kinematic_analysis(LER_means = result_LER_means,
                                               tidy_cell_lengths = fitted_cell_lengths,
                                               meristem_size_micrometre = meristem_size)

view(final_kinematic_analysis)
```

The contents of *final_kinematic_analysis* contains the values for all kinematic parameters for each plant:

R SCRIPT-installation and demo script-... * | final_kinematic_analysis | fitted_cell_lengths | result_LER_me

Filter

	plant_id	LER	meristem_size	length_of_growth_zone	length_of_elongation_zone	mature_cell_length
1	C.1	3.145833	12423	72	59.577	135.7822
2	C.10	3.187500	15500	82	66.500	135.2849
3	C.2	3.208333	14792	66	51.208	120.2800
4	C.4	3.291667	12350	62	49.650	122.5998
5	C.7	3.125000	14568	71	56.432	132.8222
6	C.8	3.312500	16250	68	51.750	127.9219
7	M.10	2.375000	11675	60	48.325	126.9704
8	M.11	2.541667	13000	63	50.000	132.7221
9	M.2	2.520833	10901	59	48.099	120.5361
10	[illegible]	[illegible]	[illegible]	[illegible]	[illegible]	[illegible]

Showing 1 to 12 of 18 entries, 16 total columns

Pay attention to the leaf elongation rates. In the results from the kinematic analysis function, the values of only the plants on which a microscopy study was performed are presented, while most often also additional leaf elongation rates are available for plants that have grown to obtain the final leaf length. All mean LERs for each plant are available in the previously obtained *result_LER_means*:

demo script-... * | final_kinematic_analysis | fitted_cell_lengths | result_LER_means

Filter

	plant_id	mean_plant_LER
1	C.1	3.145833
2	C.10	3.187500
3	C.11	3.145833
4	C.2	3.208333
5	C.3	3.416667
6	C.4	3.291667
7	C.5	3.125000

Showing 1 to 7 of 33 entries, 2 total columns

After these five steps, the kinematic analysis is completed and data for each kinematics parameter is now available with the following units:

KINEMATICS PARAMETER	UNIT
leaf elongation rate (LER)	$mm \cdot h^{-1}$
length of the meristem	mm
length of the elongation zone	mm
length of the growth zone	mm
length cells leaving meristem	µm
mature cell length	µm
number of cells in meristem	
number of cells in elongation zone	
number of cells in total growth zone	
cell production rate	$cells \cdot h^{-1}$
cell division rate	$cells \cdot cell^{-1} \cdot h^{-1}$
relative cell elongation rate	$\mu m \cdot \mu m^{-1} \cdot h^{-1}$
cell cycle duration	h
time cells spend in the meristem	h
time cells spend in the elongation zone	h

For more information on the formulas used, we refer to formula section at the end of the manual. To use the calculated parameters outside R, the results can be exported to a tab-delimited .txt file with the following code:

```
# DONE
#' All calculation involved are done. You can opt to write the results to a
#' txt-file:
write_tsv(final_kinematic_analysis, "kinematic_analysis_results.txt")
```

This command generates a txt file (kinematic_analysis_results.txt) with the values is now available in the working directory:

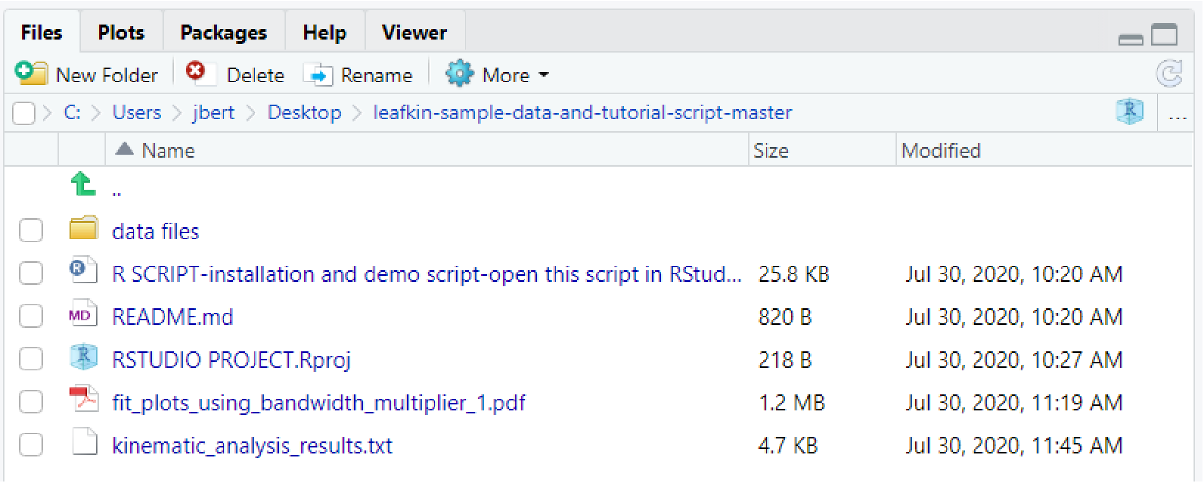

Additional code

In the tutorial script, also code to inspect all the individually calculated LERs and to recreate the cell length plot is available. Feel free to play around with this code to familiarize yourself with these types of functionality in R.

Errors and troubleshooting

Known errors of difficulties users might encounter are listed here below. An updated version of this list is maintained on the GitHub leafkin repository: https://github.com/impres-lab/leafkin. The known difficulties and errors upon the release of *leafkin* are presented here below:

Installation of *leafkin*.

- Starting with R 4.0.0 (released April 2020), R for **Windows** uses a new toolchain bundle called Rtools40. Rtools40 is advised for *leafkin* installation through GitHub on Windows and can be downloaded from https://cran.r-project.org/bin/windows/Rtools/.

Extra columns could be added through the use of Excel when creating the .txt file.

The use of Excel to enter data can, whilst being convenient, sometimes be the cause of errors in R. Excel sometimes adds extra tabs, resulting in extra rows without headers and data. These extra columns get named by R with default names, usually starting with an X. The *leafkin* functions rely on datasets with the right format. Therefore, these extra columns will result in an error when running the *leafkin* functions. A way to solve this issue is to select only the columns you are interested using the select function, provided by the dplyr package of the tidyverse. The following line will for instance only select 6 columns, starting with the first one, up until the sixth:

```
leaf_length_measurements %>% select(1:6)
```

If you make sure that the selected columns contain your data and not any extra column, processing of the data should now be errorless.

Error when creating the pdf containing cell length fit plots: cannot open file

This means that you have a pdf opened with exactly the same name as the one the *get_pdf_with_cell_length_fit_plots()* function is trying to create. This happens for instance when you create a pdf file with the *get_pdf_with_cell_length_fit_plots()* function, open the created pdf and run the function again with the pdf file still open. With the pdf file still open, R cannot replace the old file by the new file. Just close the pdf file and you should be able to run the function again.

Error when fitting cell lengths related to gridsize: Binning grid too coarse for current (small) bandwidth.

There are limits to the interval which can be chosen. An interval that is too coarse will result in an error related to the gridsize. Very small intervals will slow down the function. In our experience, the 10-centimetre growth zone of a maize leaf is ideally analysed with an interval of 0.1 or 0.01 centimetre (i.e. resulting in 101 or 1001 datapoints respectively). It is also important to note that an insufficient number of cell length measurements could result in a failed bandwidth calculation (though tests revealed that only extreme borderline disruptions in the data resulted in an error). In that case, cell lengths are not fitted, and a warning is printed after executing the function, indicating the number of plants for which no bandwidth could be calculated.

Additional information

Tidy and wide data

We use the concept of tidy as defined by Hadley Wickham (Wickham, 2014). In tidy data, each variable is saved in its own column and each observation is saved in its own row:

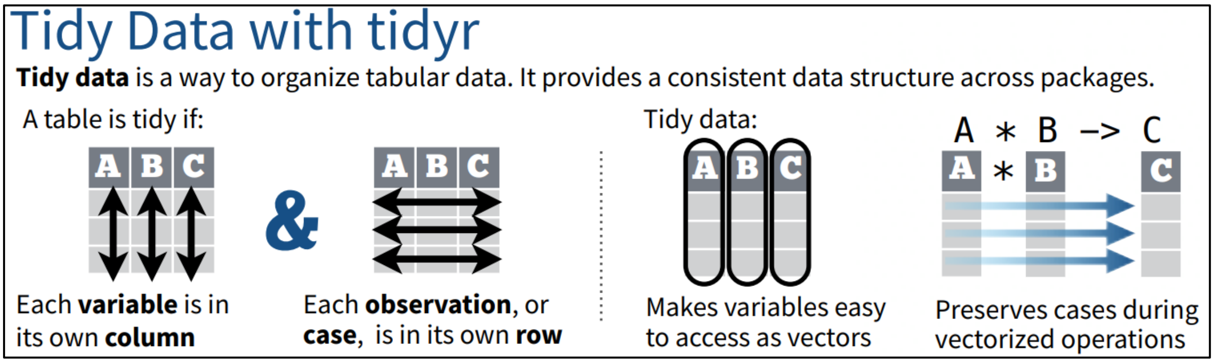

Figure 1. Snippet from the Data Import cheat sheet related to tidy data, available on: https://rstudio.com/resources/cheatsheets/.

Tidy data can be *spread* out in a wide format, where variables are spread out over multiple columns. A wide format is harder to work with in data analysis, but could be more human readable. When transforming the wide dataset back to a tidy format, it is called *melting* it (Wickham, 2014).

As an example, we will demonstrate tidy and wide tables with daily leaf length measurements. When taking measurements, one could opt to note them down in a wide format (which is often more convenient for humans):

plant_id	2020/07/20 10:40	2020/07/21 9:43	2020/07/22 10:12
plant1	121	153	186
plant2	93	130	169
plant3	112	146	180

Yet, the same data could also be presented in a tidy format, where each variable is in its own column and each observation is in its own row:

plant_id	date	leaf_length
plant1	2020/07/20 10:40	121
plant1	2020/07/21 9:43	153
plant1	2020/07/22 10:12	186
plant2	2020/07/20 10:40	93
plant2	2020/07/21 9:43	130
plant2	2020/07/22 10:12	169
plant3	2020/07/20 10:40	112
plant3	2020/07/21 9:43	146
plant3	2020/07/22 10:12	180

For data analysis, tidy date is easier to handle, since the user can now work easily with the dates and leaf lengths. The user can now also access each measurement individually with all measured variables linked to it in the same row.

Tibbles

Tibbles are an updated version of data.frames and are a way to story data as a table in R. For more information on tibbles, please visit: https://tibble.tidyverse.org/.

Formulas used by *leafkin*

Below we present the formulas used by the *leafkin* functions:

$$LER = \frac{LL_2 - LL_1}{t_2 - t_1} \qquad (1)$$

Leaf elongation rate formula (*LER*), with LL_x being the leaf length measurement x (in mm) and t_x being the time at which LL_x was taken (in data time format yyyy/mm/dd hh:mm(:ss)).

$$CCN_p = CCN_{p-1} + \frac{position_p - position_{p-1}}{\left(\frac{cell\ length_{p-1} + cell\ length_p}{2}\right)} \qquad (2)$$

Cumulative cell number (*CCN*) is a formula used internally by the kinematic analysis function to determine the cumulative number of cells at every position (*p*) by adding the cumulative cell number in the previous interval to the estimated number of cells in the current interval. The number of cells in the current interval uses the current and previous position to determine the size of the interval, which is divided by the average cell length in this interval (calculated by using the current and previous cell size).

$$P95_p = 0.95 \times \frac{\sum_{i=p}^{n} cell\ length_i}{n-p} \qquad (3)$$

The 95-percent value (P95) formula is used internally by the kinematic analysis function at every position (p) and multiplies 0.95 by the mean cell length for cell sizes starting at the current position up until the last determined cell length (n).

$$L_{gz} = \min\left(position_{(cell\ size > P95_{cell\ size})}\right) \qquad (4)$$

The growth zone size (L_{gz}) is the first position where cell size exceeds the 95-percent value of the cell sizes.

$$l_{mat} = \frac{\sum_{i=p}^{n} cell\ length_i}{n-p} \qquad (5)$$

The mature cell length (l_{mat}) is the average cell length between the first position after the growth zone (p) and the final measurement (n).

$$P = \frac{LER}{l_{mat}} \qquad (6)$$

The cell production rate (*P*) is calculated by dividing the leaf elongation rate (*LER*) by the mature cell length l_{mat}.

$$N_{mer} = CCN_i + \left((CCN_{i+1} - CCN_i) \times (L_{mer} \bmod interval\ size)\right) \qquad (7)$$

The number of cells in the meristem (N_{mer}) is determined by the cumulative cell number (CCN) at position *i*, where *i* is the last position which is still located within the meristem size, plus the difference in CNN between position *i* and *i+1*, multiplied by the meristem length (L_{mer}) modulo interval size.

$$l_{mer} = cell\ length_i + \left((cell\ length_{i+1} - cell\ length_i) \times (L_{mer} \bmod interval\ size)\right) \qquad (8)$$

The length of the cells leaving the meristem (l_{mer}) is determined by the cell length at position *i*, where *i* is the last position which is still located within the meristem size, plus the difference in cell length between position *i* and *i+1*, multiplied by the meristem length (L_{mer}) modulo interval size.

$$N_{gz} = CCN_i \qquad (9)$$

The number of cells in the growth zone (N_{gz}) is equal to the cumulative cell number at position *i*, where *i* is the position at which the length of the growth zone L_{gz} was determined.

$$N_{el} = N_{gz} - N_{mer} \qquad (10)$$

The number of cells in the meristem (N_{mer}) is determined by subtracting the number of cells in the growth zone (N_{gz}) by the number of cells in the meristem (N_{mer}).

$$D = \frac{P}{N_{mer}} \qquad (11)$$

The average cell division rate (*D*) is determined by dividing the cell production rate (*P*) by the number of cells in the meristem (N_{mer}).

$$T_c = \frac{\ln 2}{D} \qquad (10)$$

The cell cycle duration (T_c) is determined by dividing the natural logarithm om 2 by the average cell division rate (*D*).

$$T_{el} = \frac{N_{el}}{P} \qquad (11)$$

The time in the elongation zone (T_{el}) is determined by dividing the number of cells in the elongation zone (N_{el}) by the cell production rate (*P*).

$$T_{div} = \log_2 N_{mer} \times T_c \qquad (12)$$

The time cells spend in the meristem (division zone, T_{div}) is determined by the log2 of the number of cell in the meristem (N_{mer}) multiplied by the cell cycle duration (T_c).

$$R_{el} = \frac{\ln l_{mat} - \ln l_{div}}{T_{el}} \qquad (13)$$

The relative cell elongation rate (R_{el}) is determined by the difference in the natural logarithm of mature cell length (l_{mat}) and the natural logarithm of the lengths of the cells leaving the meristem (l_{div}), divided by the time cells spend in the elongation zone (R_{el}).

References

Bertels J, Huybrechts M, Hendrix S, Bervoets L, Cuypers A, Beemster GTS. Cadmium inhibits cell cycle progression and specifically accumulates in the maize (*Zea mays* L.) leaf meristem. Journal of Experimental Botany, **in press**.

RStudio Team. 2015. RStudio: Integrated Development for R. RStudio, Inc.

Sprangers K, Avramova V, Beemster GTS. 2016. Kinematic Analysis of Cell Division and Expansion: Quantifying the Cellular Basis of Growth and Sampling Developmental Zones in *Zea mays* Leaves. Journal of Visualized Experiments, 1–11.

Wickham H. 2014. Tiday Data. Journal of Statistical Software **59**, 1–23.
